# Asthma in Pregnancy: Pathophysiology, Diagnosis, Whole-Course Management, and Medication Safety

**DOI:** 10.1155/2020/9046842

**Published:** 2020-02-22

**Authors:** Huijie Wang, Na Li, Huaqiong Huang

**Affiliations:** Department of Respiration and Critical Care Medicine, The Second Affiliated Hospital of Zhejiang University School of Medicine, Hangzhou 310000, China

## Abstract

Asthma in pregnancy is a health issue of great concern. Physiological changes and drug compliance during pregnancy can affect asthma control in varying degrees, and the control level of asthma and the side effects of asthma medications are closely related to the adverse perinatal outcomes of mother and fetus. This article provides an update on the available literature regarding the alleviating or aggravating mechanism of asthma in pregnancy, diagnosis, disease assessment, and systematic management, to provide a new guidance for physician, obstetric joint doctor, and health care practitioner.

## 1. Introduction

Asthma in pregnancy is a common respiratory disease, and the burden of it has gradually increased worldwide, which has become one of the most common public health problems. The incidence of asthma in pregnancy increases from 3% to 8% in the United States after 1994 [1] and 8% of women suffer from asthma during pregnancy in Britain [2] and 12% in Australia [3]. Asthma control levels often change during pregnancy. It is generally believed that one-third of asthma patients are aggravated due to pregnancy, and most occur in the middle of pregnancy; one-third improved, and no significant changes are observed in the remaining 1/3 of patients. But a latest multicase-control study shows that the percentage of asthma worsening during pregnancy is 18.8%, lower than the previous data, and the worsening is significantly associated with the severity of the disease [4]. There are also many problems in the control of asthma during pregnancy. Studies show that about 65% of patients have poor control of asthma during pregnancy, inhaler technology is not correct in 64.4% of cases, only 38% of patients know the difference between asthma reliever and controlled medications, 12.7% of patients receive a written asthma action plan, 17% of patients have spirometry in the past 5 years, and 3.8% of them have peak expiratory flow meter at home [5]. Studies have shown that maternal asthma increases the risk for adverse complications in fetuses and mothers, including SGA (small for gestational age), LBW (low birth weight), congenital malformations (cleft lip or cleft palate), increased perinatal mortality, PB (premature birth), maternal preeclampsia, gestational hypertension, gestational diabetes, prenatal hemorrhage, caesarean section, urinary tract infection, excessive amniotic fluid, and premature rupture of membranes, especially for those patients with severe or uncontrolled asthma during pregnancy [6, 7].

## 2. Mechanisms of Asthma Remission or Onset during Pregnancy

The pathogenesis of asthma remission or aggravation during pregnancy is related to the physiological or pathological changes caused by pregnancy, mainly including the mechanical changes caused by uterine enlargement and the direct or indirect effects of hormonal changes during pregnancy.

With the increase of uterus and abdominal pressure, the diaphragm is elevated by 4-5 cm, subcostal angle increased 50% (68° to 103° from early to late pregnancy), and the transverse and anteroposterior diameter of thoracic increased. The above changes are partially compensated by relaxation of ligamentous attachments of the ribs which leads to the decrease of the thoracic compliance. As a result, the total lung volume decreases by 5% and FRC (functional residual capacity) decreased by 20% [8]. Moreover, the increased body weight leads to larger neck circumference and smaller oropharyngeal area which contributes to dyspnea during pregnancy [9].

During pregnancy, in order to meet the needs of maternal and fetal metabolism, a series of important changes occur in hormone levels, including the obvious increase of progesterone, estrogen, cortisol, and prostaglandin, all of which have different effects on the course of asthma.

Progesterone is a stimulant of respiratory dynamics, which can increase the sensitivity of respiratory center to carbon dioxide, while estrogen can increase the sensitivity of progesterone receptor in respiratory center and jointly participate in the change of respiratory function [10]. The minute ventilation increases by 30%–50% which is mainly due to a 40% increase in tidal volume, while there is no significant change in respiratory rate. TLC (total lung capacity), VC (vital capacity), lung compliance, and DLCO (diffusion capacity) remain unchanged.

FVC (forced vital capacity), FEV1 (forced expiratory volume in 1 s), the ratio of FEV1 to FVC, and PEFR (peak expiratory flow rate) have no significant changes during pregnancy compared with nonpregnancy [8]. Therefore, spirometry can be used to identify dyspnea in normal pregnancy and reflect the changes in respiratory diseases. In addition to acting on the respiratory center, progesterone can mediate mucosal vasodilation and congestion, resulting in the increase of pregnancy rhinitis and epistaxis incidence [11] and oropharyngeal and laryngopharyngeal airways that contribute to the attack of asthma in pregnancy.

Estradiol can increase maternal innate immunity and cell- or humoral-mediated adaptive immunity. Low concentration of estradiol can promote CD4+Th1 cell response and cell-mediated immunity. High concentration of estradiol can enhance CD4+Th2 cell response and humoral immunity. Progesterone inhibits the maternal immune response and changes the balance between Th1 and Th2 responses. Although cell-mediated immunity is more important in respiratory viral infections, the transfer of Th1 to Th2 immunity is considered to be an important mechanism for asthma induced by hormones during pregnancy [12, 13].

Women are in the state of hypercortisonism during pregnancy; meanwhile, the placenta secretes both CRH (corticotropin-releasing hormone) and ACTH (adrenocorticotropic hormone), which results in the increase of free cortisol and conjugated cortisol during pregnancy. The increased free cortisol mediates the increase of beta-adrenoceptor and the enhancement of bronchiectasis [9]. Increased secretion of prostaglandin E2 (PGE2) during pregnancy, through anti-inflammatory, inhibition of smooth muscle cell proliferation, bronchial relaxation, and other mechanisms, has a protective effect on the incidence of asthma. In addition, progesterone also has the effect of changing the airway smooth muscle tension and causing bronchiectasis [10]. These factors are associated with asthma remission during pregnancy.

Generally speaking, the effects of mechanical and biochemical changes on the respiratory system of pregnant are very complex, especially the effects of various hormones on the respiratory center, peripheral airway, and immune system, leading to nonasthmatic pregnant women experiencing varying degrees of dyspnea during pregnancy. For pregnant women with asthma, it is very important to strengthen the management of asthma during pregnancy to avoid maternal hypoxia and maintain adequate fetal oxygenation.

## 3. Diagnosis of Asthma in Pregnancy

General asthma is defined by the history of more than one type of respiratory symptoms such as wheeze, shortness of breath, chest tightness, and cough that vary over time and in intensity, often appear or worsen with viral infections, and attack at night or on waking, usually triggered by exercise, laughter, allergens, and cold air, together with variable expiratory airflow limitation [14]. If one of the tests is positive including bronchodilator reversibility test, bronchial provocation tests, and PEF variability, it can confirm variable expiratory airflow limitation.

Compared with general asthma, asthma in pregnancy has similar clinical manifestations. However, if a pregnant woman only complains of shortness of breath or chest tightness, we must be careful to diagnose based on her medical history. As we know, over two-thirds of pregnant women experience some form of shortness of breath or chest tightness during the gestation period because of physiological changes of pregnancy [11]. In addition, it would not be advisable to carry out a bronchial provocation test to prevent maternal hypoxia and fetal distress.

## 4. Assessment and Monitoring of Asthma in Pregnancy

Assessment of asthma usually includes asthma control (both symptom control and future risk of adverse outcomes), treatment issues particularly inhaler technique and adherence, and any comorbidities that can contribute to symptom burden and poor quality of life [14]. Lung function, FeNO (exhaled nitric oxide), ACT (Asthma Control Test) scores [15], and blood eosinophil counts are important tools for asthma assessment. PEFR is used to monitor lung function, which should be 380 to 550 L/min, but the personal best value is required. Maintaining 80–100% of personal best value is considered normal range for asthma patients. FeNO is an indicator of airway inflammation; studies have found that pregnant women with asthma have the same FeNO as before, which is associated with asthma control [16–18]. Adjusting treatment of asthma in pregnancy according to FeNO can reduce acute attacks and neonatal admission rate.

The assessment of asthma in pregnancy is a collaborative effort by physician, obstetric joint doctor, and health care practitioner. It is necessary not only to evaluate pregnant women but also to comment on the growth and development of the fetus. And the frequency of assessments needs to be more frequent, once a month is recommended by GINA (Global Initiative for Asthma).

## 5. Treatment and Systemic Management of Asthma in Pregnancy

Whole-course management of asthma in pregnancy can reduce the negative effects of fluctuations in asthma symptoms or acute exacerbation on pregnant women and the fetus. The GINA guidelines suggest that poor asthma control and acute exacerbations during pregnancy are more risky than taking asthma medications. The long-term goals of asthma management are to achieve good symptom control, maintain normal activity levels, and minimize the risk of acute attacks, irreversible damage to lung function, and drug-related adverse effects. For asthma in pregnancy, it is also essential to avoid side effects of drugs on pregnant and fetuses and give birth to healthy babies. The principle of asthma treatment is defined as the selection of appropriate treatment based on the severity and control level of patient. A written asthma control plan should be developed for each newly diagnosed patient, regular follow-up and monitoring, and adjusting treatment according to patient control level to achieve and maintain asthma control.

### 5.1. Pharmacologic Therapy

Though the principles of medication for asthma in pregnancy are similar to those of nonpregnant patients, there are differences between them in the implementation of stepwise approach. For general asthma, the control level of asthma is assessed at 3 months after the use of controller medication. Those with good asthma control are treated with step down treatment, while those with persistent asthma symptoms or acute exacerbation are treated with step-up treatment. For asthma in pregnancy, the GINA guidelines and the Australian Asthma Handbook recommend that asthma control levels should be assessed monthly. Step-down treatment is not a priority in order to avoid acute exacerbation of asthma unless the pregnant woman's drug dosage is inappropriate [14, 19]. In addition, anti-IgE monoclonal antibodies and specific immunotherapy should not be initiated during pregnancy [20]. Currently, the most commonly used and safe drugs during pregnancy include glucocorticoids, beta2-agonist, anticholinergics, theophylline, leukotriene receptor antagonists (LTRAs), omalizumab, and allergen immunotherapy (AIT).

#### 5.1.1. Glucocorticoids

Among glucocorticoids, inhalation administration is predominant. Inhaled glucocorticoids (ICS) can effectively inhibit the number and activity of inflammatory cells in the airway; moreover, it can significantly reduce the side effects of systemic medication since its effect on local airway. Budesonide is the most commonly used and safe ICS during pregnancy.

A study based on Danish medical registries, of 83043 primiparous women who gave birth to a live-born singleton in 1999–2009, has found no association between use of glucocorticoids (both ICS and oral glucocorticoids, OCS) in early pregnancy and risk of oral clefts or congenital malformations overall in the offspring [21]. Tegethoff et al. have studied 4083 mother-child pairs from the Danish National Birth Cohort and found that ICS for asthma treatment during pregnancy (*n* = 1231; 79.9% budesonide, 17.6% fluticasone, 5.4% beclomethasone, and 0.9% other or unspecified glucocorticoids) is not associated with offspring disease risk in most categories, except for offspring endocrine, metabolic, and nutritional disorders [22]. However, Mortenet al. have indicated that fraction of exhaled nitric oxide- (FENO-) guided asthma management during pregnancy reduces doctor-diagnosed asthma in the offspring at the stage of preschool, partly mediated through alterations in use and dosing of ICS during the trial of managing asthma in pregnancy [23].

Many studies have failed to clarify whether the use of ICS increases the risk of teratogenic, but it is clear that asthma patients who do not use ICS have a significantly increased risk of giving birth to low-weight children, suggesting that the risk of poor asthma control is much higher than medication [24]. Vicki et al. support this conclusion. Their research suggests that the placenta plays an important role in regulating fetal growth by producing an enzyme, 11*β*-hydroxysteroid dehydrogenase-2 (11*β*-HSD-2), which can convert endogenous and exogenous glucocorticoids derived from the maternal circulation to their inactive metabolites. As a consequence, 11*β*-HSD-2 activity has a positive correlation with birth weight. Asthmatic patients without using ICS and 11*β*-HSD-2 activity are reduced in placentae of female fetuses and that may be associated with inflammatory factors produced during the pathogenesis of asthma. While using low, moderate, or high doses ICS will not affect placental capacity to metabolise glucocorticoids, so it does not affect the birth weight, which may be related to the fact that ICS is able to increase production of placenta 11*β*-HSD-2. Additionally, uncontrolled asthma frequently shows associations with adverse perinatal outcomes, as previously demonstrated with exacerbations in cohorts of asthma in pregnancy, and the continued use of ICS with budesonide is recommended and has a particularly good safety profile [25]. In short, asthma itself has more adverse effects on the placenta than its treatment [26].

In addition, studies have shown that low doses of ICS in patients with asthma during pregnancy can reduce the risk of acute exacerbation and readmission, but there are safety issues with high-dose ICS. Lucie Blais et al. have conducted a cohort study of 13280 pregnancies of women with asthma (1990–2002) and found that women who use >1000 *μ*g/day ICS are significantly more likely to have a baby with a malformation than the women who use >0 to 1000 *μ*g/day, while women who use >0 to 1000 *μ*g/day are not found to be more at risk than women who do not use ICSs during the first trimester [27]. A cohort study incorporating 7376 pregnancies also discovers no increased prevalence of LBW, PB, and SGA in pregnant women with asthma for long-acting beta2-agonists (LABA) use and ICS doses <125 *μ*g/day, while a trend for an increased prevalence of the outcomes is seen for ICS doses above 125 *μ*g/day. Maternal glucocorticoid-regulated systems are susceptible to ICS (800 *μ*g/day for first and second trimester and 900 *μ*g/day for third trimester) for asthma during pregnancy, but glucocorticoid-regulated pathways in the fetus are not affected by ICS use, which implied that ICS use for the control of asthma during pregnancy is unlikely to contribute to adverse effects on fetal growth and development [28].

OCS is sometimes necessary depending on the severity of asthma. But we should keep in mind that, among all OCS, prednisone, prednisolone, and methylprednisolone can cross the placenta at very low concentrations, while dexamethasone and betamethasone reach the fetus at higher concentrations. Among them, prednisone is the most common OCS. Before entering the fetal blood circulation through the placenta, 87% of blood concentration is inactivated by the action of 11*β*-HSD-2, with little impact on the fetus.

Accumulating evidence suggests that the use of OCS can increase the incidence of cleft lip and palate in the fetus, especially in the first trimester of pregnancy. The incidence of cleft lip and palate in general population is 0.1%, while in oral glucocorticoids-taking pregnant women it is 0.3% [29]. However, there is also study which believed that the risk of cleft palate is uncorrelated with the application of OCS during pregnancy [30]. And using OCS throughout pregnancy may increase the incidence of preeclampsia, preterm birth, and low birth weight [29]. An animal experiment shows that glucocorticoid exposure in early placentation could contribute to preeclampsia, with the mechanisms involving inhibition of trophoblast proliferation, migration, invasion, and epithelial-mesenchymal transition by glucocorticoid [31]. In population-based studies, OCS has also been reported to increase the risk of preeclampsia [32]. Jennifer et al. have conducted a meta-analysis on papers from 1975 to 2012 which are related to the risk of adverse outcomes in neonates with acute attack of asthma, oral corticosteroid, or asthma severity, which shows that acute attack of maternal asthma and oral corticosteroid use have a significant effect on low birth weight and preterm delivery; moderate-to-severe asthma during pregnancy is also associated with an increased risk of SGA and low birth weight infants [33]. There is evidence that asthma in pregnancy perceives a higher risk of OCS medication use on the fetus, compared with ICS treatment, which may lead to abnormal behavior of women during pregnancy [34]. In terms of the risk tradeoffs between poor asthma control and the use of OCS, the current consensus remaining is that the risk of inadequate treatment is higher than the potential risk of systemic glucocorticoids.

#### 5.1.2. Beta2-Agonist

Beta2-agonists are suitable for asthma patients of all levels during pregnancy, and quantitative inhalation or atomization of solution is the main administration method. Short-acting beta2-agonist (SABA) including salbutamol, terbutaline, and pirbuterol is used as reliever medications. NAEPP recommended that the preferred choice in SABA is salbutamol because of its safety data for pregnant with asthma. LABA, salmeterol, and formoterol are as controlled medications. LABA is similar to salbutamol in pharmacologic and toxicologic profile; while there are limited data on their use during pregnancy, salmeterol might be chosen which has been available longer [29]. Report indicates that using of long-acting beta2-agonists (LABA) during pregnancy will not increase the prevalence of the perinatal outcomes [35]. However, in the current studies, the relationship between beta2-agonists and perinatal adverse outcomes is still controversial.

A research finds no significant relationships between major congenital malformations and SGA infants with exposure to beta2-agonists, theophylline, cromolyn, and corticosteroids during the first trimester or at any time [36]. A retrospective cohort study of 13117 pregnancies finds no increased risk of congenital malformations with SABA exposure during the first trimester, while using LABA in the first trimester is significantly associated with a higher risk of major “cardiac malformations” and major “other and unspecified congenital malformations.” However, bias due to different asthma severity or chance should be considered [37]. A review identified 21 original studies; 4 studies report that use of SABA during pregnancy increases risk of congenital malformations, while one study reports that high doses of SABA decrease the risk. Four studies indicate that beta2-agonists (SABA and/or LABA) contribute to increased risk of congenital malformations; one study shows a significant increased risk with LABA. One study shows that LABA led to low birth weight babies [38]. A population-based case-malformed control study including 76,249 registrations of congenital anomalies from 13 EUROmediCAT registries finds that exposure to first trimester with inhaled beta2-agonists increases the risk of cleft palate and gastroschisis. Also, exposure to the combination of LABA and ICS is associated with renal dysplasia [39]. However, in the cohort study of 567 cases of hypertensive disorders of pregnancy and 256 cases of preeclampsia/eclampsia, LABA use is not associated with increased risks of HDP or preeclampsia/eclampsia, suggesting the safety of LABAs for the cure of asthma in pregnancy [40]. Besides, maternal asthma is closely associated with increased risk of offspring autism spectrum disorder (ASD), but this adverse effect is not associated with prenatal exposure to asthma medications including LABA [41]. GINA (2019) suggests that SABA alone is no longer as the recommended therapeutic strategy for asthma; instead GINA recommends that all adults and adolescents with asthma should receive either symptom-driven (for mild asthma) or daily inhaled anti-inflammatory controller treatment, to reduce their risk of serious exacerbations and to control symptoms.

#### 5.1.3. Anticholinergics

Anticholinergics include the short-acting muscarinic antagonist (SAMA), ipratropium bromide, and long-acting muscarinic antagonists (LAMAs), tiotropium bromide. GINA guidelines suggest that combination of formoterol and SABA could be used to treat severe acute asthma attacks. Previous studies also have demonstrated that the use of SABA plus ipratropium bromide can effectively improve severe acute asthma control. However, there is still much controversy over whether ipratropium bromide can reduce the admission rate of patients and improve their lung function [42]. A study finds that, for patients with poor asthma control by applying ICS or ICS combined with LABA, tiotropium as add-on therapy can improve pulmonary function and reduce the frequency of exacerbations [43]. In a systematic review of 13 randomized-placebo controlled trials, the use of anticholinergic tiotropium ameliorates asthma control in patients with moderate symptomatic asthma who have already received medium-to-high doses ICS or ICS/LABA [44], while there are few studies on the use of anticholinergics in asthma patients during pregnancy. More studies are needed to confirm the relationship between anticholinergic drugs and perinatal adverse outcomes.

#### 5.1.4. Theophylline

Low-dose theophylline is considered as an alternative for mild persistent asthma during pregnancy and as an alternative adjunctive long-acting bronchodilator therapy for moderate-to-severe asthma when ICS alone cannot provide adequate control of patient's asthma [45]. Theophylline can pass through the placental barrier. There is no significant difference in theophylline concentration between maternal and umbilical cord serum, when the levels of theophylline are greater than 10 *μ*g/ml, transient neonatal vomiting, tremor, and tachycardia can occur [46]. The blood concentration should be maintained at 5–15 *μ*g/ml in nonpregnant asthma patients and 5–12 *μ*g/ml in pregnant women [47]. When serum theophylline is higher than 20 *μ*g/ml, the risk of theophylline toxic reactions will increase [46]. Since the recommended dose of theophylline is close to the toxic dose, the plasma or urine concentration must be closely monitored during the treatment, especially for pregnant women whose liver metabolic function decreased. The updated GINA guidelines indicate that low-dose theophylline (serum concentrations must be monitored closely) is considered as an alternative, but not a preferred treatment for mild persistent asthma during pregnancy. For moderate-to-severe asthma during pregnancy, theophylline may be considered as an alternative adjunctive long-acting bronchodilator therapy but not a preferred therapy when ICS alone does not provide adequately asthma control during pregnancy. Additionally, theophylline use has no or minimal effects on fetal growth and reduces perinatal complications when maternal asthma is adequately controlled [45]. In a multicenter prospective study, compared with the effects of ICS and oral theophylline on asthmatic pregnant women, the two cohorts have similar rates of asthma exacerbations, asthma outcomes, and obstetric and perinatal outcomes, while theophylline group has more adverse events, drug withdrawal rate during observation period and FEV1 <80% predicted value of lung function than glucocorticoids group [48]. At present, the use of controlled-release theophylline preparations is advocated, which can dilate bronchus for 10–12 hours, and is conducive to the control of night asthma. Intravenous aminophylline is mostly used in acute asthma attacks, and no teratogenic effect has been found.

#### 5.1.5. Leukotriene Receptor Antagonists (LTRAs)

Antileukotrienes are divided into two categories: LTRAs including zafirlukast and montelukast and 5-lipoxygenase pathway inhibitors, zileuton. Among them, zileuton is not recommended for use in pregnancy because of the lack of relevant research and the need to monitor liver function during medication. LTRAs alone are less effective compared with ICS and they are generally taken in combination with other asthma medications. However, the use of LTRAs in combination with ICS has the same effect as those of LABAs in combination with ICS in steroid-naïve asthmatic patients [49]. With the exposure to LTRAs in asthma patients during pregnancy, no increased risk for major birth defects is observed in a cohort study of 1164 first-trimester-exposed pregnancies [50]. And adverse outcomes including pregnancy loss, gestational diabetes, preeclampsia, low maternal weight gain, preterm delivery, low Apgar scores, or small size are discovered in a cohort study of 96 infants treated with leukotriene receptor antagonists in pregnancy [51]. The LTRAs are only recommended for patients who had a favorable response to them before becoming pregnant. They are an alternative to ICSs and are not preferred as a treatment option in mild persistent asthmatics during pregnancy [45, 52].

#### 5.1.6. Omalizumab

Anti-IgE monoclonal antibody omalizumab is as an add-on therapy for the treatment of nonpregnant patients with moderate-to-severe persistent asthma that is inadequately controlled with ICS and has the effect of preventing exacerbation, reducing the frequency of asthmatic symptoms, reducing the frequency of emergency room visit or hospital admission, and reducing the steroid dose. A prospective, observational study, EXPECT (the omalizumab pregnancy registry), includes 191 pregnant women who exposed to ≥1 dose of omalizumab within 8 weeks prior to conception or at any time during pregnancy and collected data on 169 pregnant women at the time of data cut-off. The proportions of major congenital anomalies (4.4%), prematurity (14.5), low birth weight (3.2%), and SGA (10.9%) observed in the EXPECT registry are consistent with findings from other studies in this asthma population. The prevalence of major congenital defects in EXPECT is no higher than those reported in the general population with asthma. Omalizumab does not appear to increase the risk of prematurity or SGA infants beyond that seen in general asthma population [53]. While considering the possible anaphylaxis, this therapy is not initiated in pregnancy though sometimes may be continued if already in progress [54].

In addition to the safety, there is still a notable problem, namely, the dose titration of omalizumab. Omalizumab should be dosed according to body weight and pretreatment serum total IgE levels and is injected subcutaneously once every 2 or 4 weeks. Each injection ranges from 75 to 600 mg, and the maximum dose is no more than 375 mg every 2 weeks in the United States of America and 600 mg in the European Union. In the European Union, the eligible baseline IgE level is 30–1500 IU/ml. In all countries, body weight must be no more than 150 kg. Accordingly, patients whose body weight or baseline IgE levels are not in the above range should not receive the treatment [55]. For pregnant women with asthma, maternal weight changes persistently over a short period of time as the fetus grows; therefore, the method of adjusting the amount of omalizumab according to body weight is a challenge for maternal asthma.

After the use of high-dose ICS, approximately 40–50% of uncontrolled asthma patients still have persistent airway eosinophilia. Accumulating evidence suggests that interleukin- (IL-) 5 contributes to airway eosinophilic inflammation. In addition to omalizumab, another two anti-IL-5 monoclonal antibodies, mepolizumab and reslizumab, have also been approved as maintenance treatment program for patients with uncontrolled, persistent eosinophilic asthma with an exacerbation phenotype. Mepolizumab has been approved by FDA for maintenance therapy of severe asthma patients aged 12 or older and can reduce exacerbations by approximately 50% in patients with severe asthma [56]. And reslizumab is also FDA approved for maintenance therapy of severe asthma in patients aged 18 or older [57]. Benralizumab, another humanized monoclonal antibody selective for IL-5 receptor, has also been approved by FDA for add-on maintenance treatment of severe asthma in patients aged 12 or older with an eosinophilic phenotype. There are no adequate studies of monoclonal antibodies such as mepolizumab, reslizumab, and benralizumab use in pregnant women due to the fact that monoclonal antibodies can cross the placenta during the third trimester. However, in a study of cynomolgus monkey, no harmful effects were observed on the fetus or on neonatal growth up to 6.5 months after birth when monkeys were given IV benralizumab every 2 weeks which is the maximum recommended human dose throughout pregnancy [58]. Collectively, there is rarely report on the use of monoclonal antibodies against IL-5 in asthma during pregnancy and their adverse effects on the maternal health or the development of the offspring.

#### 5.1.7. Allergen Immunotherapy (AIT)

AIT is currently considered to be the only treatment for the etiology of asthma, which relieves asthma symptoms and reduces asthma attacks by regular subcutaneous injection, oral administration, or sublingual administration of an injection or oral preparation of known allergens. For the general asthma patients whose symptoms are well controlled or partially controlled and are clearly linked to a relevant allergen [59], it is a valuable treatment challenge to apply AIT after weighing the pros and cons in order to improve asthma symptoms, lung function, or bronchial hyperreactivity and reduce medication requirements, but for the pregnant women with asthma, we should be more cautious. A review reports that uterine cramps may occur during anaphylaxis, also there are possible side effects of venom immunotherapy, and a 28-year-old woman under wasp venom desensitization has a premature birth in her 24th week of pregnancy [60]. It is generally recognized that AIT should not be initiated during pregnancy because the risk of anaphylaxis is unknown and the benefits appear minimal. However, it can be continued in patients who already received an allergic vaccine, on a stable and nonescalating dose, and whose symptoms appear to improve [54]. A group of 121 pregnancies from 90 atopic mothers who have received immunotherapy during pregnancy are studied retrospectively. The incidence of prematurity, toxemia, abortion, neonatal death, and congenital malformation is not greater than that for the general population. It suggests that AIT can be cautiously continued during pregnancy without significant risk to either mother or fetus [61]. There are still studies concluding that AIT (sublingual or subcutaneous) is safe during pregnancy, even when initiated for the first time in a pregnant patient. A retrospective study included 81 patients (109 pregnancies) receiving subcutaneous immunotherapy and 60 pregnant patients (82 pregnancies) without receiving immunotherapy. In the treatment group, the incidence is similar to or less than the general population with regard to abortion, perinatal mortality, prematurity, toxemia, and congenital malformation. In the control group, there is a higher incidence of abortion, prematurity, and toxemia as compared with those treated with immunotherapy. In addition, seven patients of the treatment group who are already pregnant when immunotherapy is initiated did not develop any complications and all delivered normally [62]. In a prospective study, the subjects are divided into three groups: the treatment group receiving SLIT (sublingual AIT, *n* = 155) with either house dust mite or a mixture of up to five allergens, of which 24 pregnancies received sublingual immunotherapy for the first time during pregnancy, the control group A received budesonide 400 *μ*g twice daily (*n* = 85), and the control group B receives rescue treatment with salbutamol (*n* = 40). After follow-up for six years, the incidence of perinatal deaths, prematurity, and toxemia of pregnancy is less than that in the general population but the incidence of complications is higher in both control groups. None of the 24 patients develops complications during pregnancy and none has reactions to SLIT [63].

### 5.2. Acute Attack of Asthma in Pregnancy

Asthma attack in pregnancy is defined as requiring medical intervention during pregnancy, including unscheduled doctor visits, emergency department presentations, hospitalization, or requirement for OCSs, with the occurrence rate of at least 20%–45%, and 5%–11% of women suffering severe attack (requiring OCS). The risk of severe attack in patients with mild, moderate, and severe asthma is 8%, 47%, and 65%, respectively, reported by a prospective cohort study of 146 women with asthma and pregnancy. And attack during pregnancy occurs mainly in the late second trimester, a mean of 25.1 ± 0.9 weeks of gestation. The major triggers are viral infection and nonadherence to ICS; besides, the undertreatment of asthma and changes in hormone levels also play a role [64–66]. Attack of asthma during pregnancy is connected with increased risk of adverse pregnancy and perinatal outcomes. Current studies have shown that acute attack increases the risk of LBW; however, the relationship between acute attack and other adverse outcomes is still controversial, such as PB, SGA, congenital malformations, neonatal death, and hospitalizations, as well as a range of maternal and placental complications, preeclampsia, gestational diabetes, placenta previa, and caesarean section [66, 67].

For asthma management, early identification of asthma attack during pregnancy is very important. Symptoms worsening is the main manifestation of the patient, combined with PEF monitoring and attention to fetal activity which is helpful in judging the condition. If PEF decreases more than 50%, personal predicted suggests severe exacerbation [29]. Patients with asthma in pregnancy can be treated by themselves in the early stage of asthma attack, and the specific steps and medication are the same as nonpregnancy asthma. Once the patient's asthma symptoms are poor relieved or even worse, they should be hospitalized in time. In order to avoid hypoxia of pregnant women and fetuses, consider the following: giving oxygen inhalation to pregnant women, continuously monitoring oxygen saturation and fetal heart rate, using SABA and ICS actively, adding OCS if the patients are with poor effect, and even using intravenous hormone as soon as possible for the patients with respiratory failure.

### 5.3. Intrapartum Management

90% of asthmatic pregnant women will not have an acute attack during childbirth, and only inhaling bronchodilators can control symptoms, while patients with chronic or frequent use of steroids may be at risk of adrenal insufficiency during delivery, and a pressure dose (100 mg of hydrocortisone every 6–8 hours) can be used to prevent adrenal insufficiency during delivery and on the first day after delivery. Furthermore, if mothers use SABA in large doses, newborns, especially premature babies, need to be tested for blood glucose within 24 hours. In addition, asthma patients should also be cautious about using some common measures during delivery such as drugs for cervical ripening, analgesic, and anesthesia. Oxytocin and prostaglandin E2 can be to induce labor in asthmatics. Fentanyl is an acceptable drug to relieve pain; however, morphine, meperidine, or other narcotics for analgesia are likely to cause the release of histamines and bronchospasm. Epidural rather than general anesthesia is recommended for asthmatics who need a painless delivery to reduce the risk of pulmonary infection and atelectasis [68, 69].

### 5.4. Education

Patient education as a nonpharmacologic intervention is equal in importance with the pharmacologic treatment. As mentioned at the beginning of the article, poor drug compliance and incorrect use of inhaled drugs are important factors that make asthma difficult to control, especially in pregnant women, given the potential risks of drugs to mothers and fetuses. The purpose of education is to improve patients' compliance, standardize medication according to the asthma action plan, master correct inhalation techniques, and self-monitor the condition.

### 5.5. Avoiding Triggers

Asthma patients should avoid or reduce irritants such as allergens, climate change, drugs, sports, specific environments, and occupations and try specific environmental controls such as air filtration or special bed covers.

In addition to the above factors, avoiding infection is an important measure to reduce asthma attacks. Viral infection has been identified as an important trigger for asthma attacks, and the risk of viral infection in asthma during pregnancy is higher than that in nonpregnant women. A prospective cohort study of 168 asthmatic pregnant women and 117 nonasthmatic pregnant women finds that 71% of asthmatic pregnant women and 46% of nonasthmatic pregnant women have a common cold during pregnancy (incidence rate ratio: 1.77, 95% CI: 1.30–2.42, *P* < 0.0001). Among asthmatic pregnant women, rhinovirus is detected in 38.5%, coronavirus in 13.8%, influenza virus A or B in 12.3%, and enterovirus in 9.2%. One-third of patients who tested positive for respiratory virus PCR are associated with acute exacerbation of asthma, and one-third lost control of their asthma [70].

### 5.6. Treatment of Complications

Asthma patients often have comorbidities (like allergic rhinitis, sinusitis, gastroesophageal reflux, obesity, chronic obstructive pulmonary disease, bronchiectasis, obstructive sleep apnea syndrome, depression and anxiety, etc.), especially those with severe and uncontrolled asthma. In pregnant women, the prevalence of gestational rhinitis, GERD, overweight or obesity (in Europe), and depression is 22%, 30–50%, 25.6%–48.4%, and 11%, respectively [71–74]. Since these comorbidities may lead to worsening of symptoms and drug interactions, even increases the risk of perinatal adverse outcomes in pregnant women, active treatment should be given and followed.

## 6. Conclusion

For pregnant women, physical structural changes, the direct effect of hormones, and the changes of immune function induced by hormones are involved in the control of asthma; one-third of asthma patients are aggravated due to pregnancy. To minimize maternal and fetal perinatal risks, assessing patient's condition by early identification of new onset asthma in pregnancy, monitoring changes of asthma symptoms, PEF variability, FeNO, and fetal activity, education for improving patients' compliance and standardizing medication, avoiding triggers, treating complications actively, and using drug therapy by a stepwise approach are all needed to be written in asthma control plan. Many studies and guidelines support the fact that inadequate treatment has a greater impact on mothers and fetuses than potential drug side effects. Achieving a good whole-course management of asthma patients in pregnancy still requires continuous efforts of clinicians and patients themselves.
